# High-Performance Flexible Piezoresistive Sensors Based on Covalently Anchored Polypyrrole Networks on Electrospun Fibrous Membranes

**DOI:** 10.3390/polym18161937

**Published:** 2026-08-07

**Authors:** Zhifei Liang, Fangrong Tan, Xinyu Zeng, Xiao Su, Zhe Tang, Paul D. Topham, LinGe Wang, Qianqian Yu

**Affiliations:** 1 South China Advanced Institute for Soft Matter Science and Technology, School of Emergent Soft Matter, Guangdong Provincial Key Laboratory of Functional and Intelligent Hybrid Materials and Devices, South China University of Technology, Guangzhou 510640, China; 202320162670@mail.scut.edu.cn (Z.L.); 15914337639@126.com (F.T.); zengxinyu1001@163.com (X.Z.); 202321062975@mail.scut.edu.cn (X.S.); tangz9705@163.com (Z.T.); 2 Aston Institute for Membrane Excellence, College of Engineering and Physical Sciences, Aston University, Birmingham B4 7ET, UK; p.d.topham@aston.ac.uk; 3 Guangdong Basic Research Center of Excellence for Energy and Information Polymer Materials, South China University of Technology, Guangzhou 510640, China

**Keywords:** electrospun composited fibers, micro-nano structure, flexible piezoresistive sensor, in situ polymerization

## Abstract

Flexible piezoresistive sensors are highly desirable for wearable health monitoring, yet balancing ultrahigh sensitivity and wide pressure detection range is a major bottleneck restricting their applications in electronic skin and soft robots. This work constructs a hierarchical piezoresistive sensor through a simple three-step fabrication: electrospinning PVDF/PAN fiber networks, polydopamine (PDA) surface modification, and in situ polypyrrole (PPy) polymerization for conductive sensing layers. As a dual-function interlayer, PDA forms hydrogen and covalent bonds with PPy to yield uniform, firm conductive coatings. The link between PPy morphology and sensing performance is clarified by regulating polymerization parameters. At a pyrrole concentration of 3 g/L, the optimized sensor achieves a high sensitivity of 220.88 kPa^−1^ (0–10 kPa) and stable linear signals up to 1 MPa, with superior cycling durability over 5000 cycles and good biocompatibility. This scalable fabrication resolves the sensitivity–range tradeoff, promising wearable medical monitoring and human–machine interaction devices.

## 1. Introduction

Flexible sensors have attracted significant attention in recent years due to their promising applications in wearable electronics, electronic skin, and human–machine interfaces, owing to their lightweight, skin-conformal, bendable, and breathable characteristics [1,2,3]. Beyond these traditional application domains, recent advances have also demonstrated the potential of flexible sensors in emerging fields such as biomedical soft robotics and agricultural monitoring [4,5]. Generally, based on different sensing mechanisms, flexible sensors can be classified into piezoresistive, piezoelectric, capacitive, and triboelectric types [6,7,8]. Among these, piezoresistive sensors are particularly appealing because of their simple signal readout, low cost, and ease of fabrication [9]. As a critical component of flexible sensors, the sensing layer often requires microstructural design to achieve high performance, such as micropyramid [10], microdome [11], micropillar [12], animal biomimetic [13], and other microstructures [14]. However, fabricating these structures typically demands complex equipment and processing techniques, posing a significant barrier to large-scale implementation [15,16]. Therefore, developing high-performance piezoresistive sensors through simple and scalable methods remains a major challenge.

Electrospinning has emerged as a promising technique for fabricating nanofibrous sensing layers, offering advantages including facile operation, wide material compatibility, and controllable fiber morphology [17,18]. Electrospun fibers exhibit high surface area, porosity, and excellent flexibility, facilitating the simplified fabrication of microstructured sensing layers [19,20]. To construct conductive networks, prior studies have integrated various conductive materials into electrospun fibers, such as graphene [21], carbon nanotubes [22], silver nanowires [23], and MXene [24]. However, the intrinsic rigidity of these nanomaterials often results in poor adhesion to flexible matrices, compromising sensor durability. In contrast, in situ polymerization of polypyrrole (PPy) offers a promising alternative, enabling uniform growth on fiber surfaces and improving interfacial integration. For example, Su et al. [25] proposed a concise strategy for widening the conductive pathway on a biomimetic scale. They successfully prepared an s-PPG@PPy sensor by polymerizing PPy in situ on the surface of PGG, achieving a superb response time (26 ms) and high sensitivity (52.42 kPa^−1^). Chen et al. [26] reported a simple method involving the in situ grafting polymerization of PPy onto PEI fibers, achieving stronger interfacial bonding, high sensitivity (9.45 kPa^−1^), and excellent dynamic durability (~30,000 cycles). These findings demonstrate that in situ grafting polymerization effectively enhances interfacial bonding. To further enhance the adhesion of PPy nanofibers, Ren et al. [27] introduced a polydopamine (PDA) layer as an adhesive interlayer on a PU skeleton due to its excellent adhesion nature. Although extensively researched, the influence of PPy growth states on the sensing performance of electrospun PPy-based piezoresistive sensors remains underexplored. What is more, it has been found that PDA possesses abundant functional groups, which enhances the connection at the interface between the conductive filler and nanofibers [28,29].

**Figure 1:**
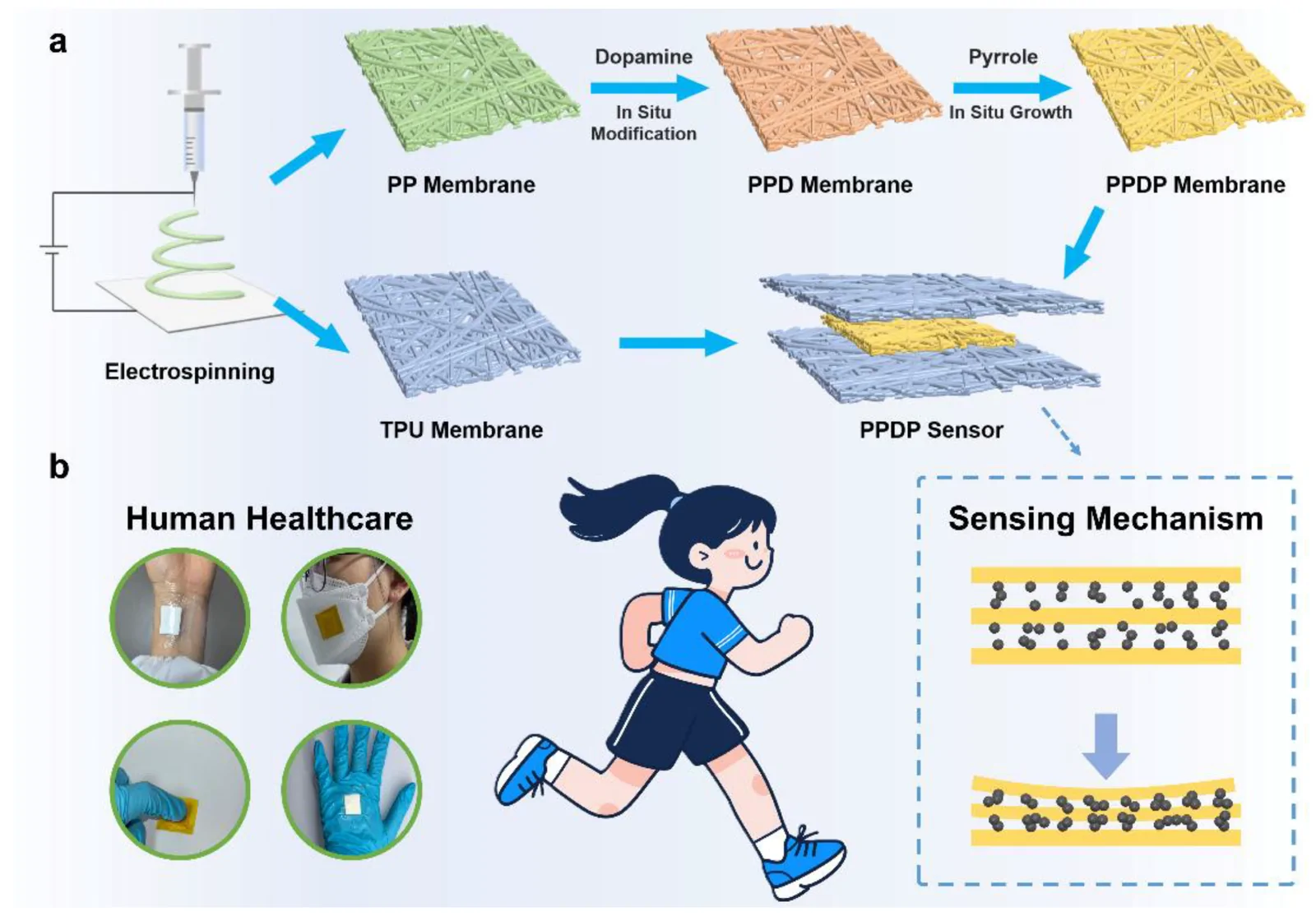
Schematic illustration of a PPDP-based piezoresistive sensor. (**a**) Preparation and mechanism of a PPDP sensor. (**b**) Application of the PPDP sensor.

In this study, we constructed a PVDF/PAN/PDA/PPy (PPDP) composite sensing layer by polymerizing PPy in situ onto PDA-modified electrospun PVDF/PAN fibers. Simultaneously, an elastic TPU/chitosan substrate was fabricated via electrospinning to encapsulate the sensing layer, resulting in a flexible piezoresistive sensor with high performance, broad sensing range, and good biocompatibility (Figure 1). Given the critical role of PPy as the conductive component, we systematically investigated how different PPy growth states affect the sensor’s performance. The optimized sensor demonstrated a maximum sensitivity of 220.88 kPa^−1^ within the pressure range of 0–10 kPa and exhibited linear response up to 1 MPa. This work provides a rapid, simple, and effective approach for fabricating high-performance flexible piezoresistive sensors, demonstrating significant potential for applications in wearable electronics, human–machine interfaces, and artificial skin.

## 2. Materials and Methods

### 2.1. Materials

Polyvinylidene fluoride (PVDF, M_w_ = 1500 kDa), polyacrylonitrile (PAN, M_w_ = 150 kDa), dopamine (DA), tris(hydroxymethyl)aminomethane hydrochloride (Tris-HCl, pH 8.5), iron(III) chloride hexahydrate (FeCl_3_·6H_2_O), and pyrrole (Py), thermoplastic polyurethane (TPU, 75A), and chitosan (CS, 90% degree of deacetylation) were obtained from Sigma-Aldrich (Shanghai, China). Tetrahydrofuran (THF) and N,N-dimethylformamide (DMF) were procured from Guangzhou Chemical Reagent Factory (Guangzhou, China). All reagents were used without further purification.

### 2.2. Preparation of the PPDP Composite Fibrous Membrane

The fabrication process of the nanofiber membranes is illustrated in Figure 1. PVDF and PAN were dissolved in DMF at a mass ratio of 2:1. This ratio was selected to balance the flexibility and piezoelectric activity of PVDF with the mechanical reinforcement provided by PAN [30]. Precursor solutions with total polymer concentrations of 10, 12, 14, and 16 wt% were then prepared for electrospinning. After stirring at 60 °C for 4 h, each solution was transferred into a 10 mL medical syringe fitted with a 21G stainless steel needle for electrospinning. The PPDP composite membrane was prepared using a handmade uniaxial electrospinning machine at room temperature [31]. During the electrospinning process, the flow rate was set at 0.5 mL/h, the applied voltage was 10.5 kV, and the distance between the needle and collector was fixed at 15 cm.

After collection, the electrospun fibrous membranes were cut into 5 cm × 5 cm pieces and immersed in a dopamine hydrochloride solution (2 g/L) prepared in Tris-HCl buffer (without additional deionized water). The membranes were allowed to react at room temperature for 12 h to obtain PVDF/PAN/PDA (PPD) membranes. Subsequently, the PPD membranes were immersed in an aqueous FeCl_3_·6H_2_O solution at 1 °C, to which pyrrole (Py) was subsequently added. The concentrations of FeCl_3_·6H_2_O and Py were systematically optimized. Specifically, FeCl_3_·6H_2_O: Py molar ratios of 1:1, 1:2, 1:3, and 1:4 were first investigated, followed by optimization of Py concentration at 1, 2, 3, and 4 g/L. The in situ polymerization was carried out at 1 °C for 12 h to yield PVDF/PAN/PDA/PPy (PPDP) membranes. Finally, the resulting PPDP membranes were removed from the solution and dried in an oven at 60 °C.

### 2.3. Characterizations

The morphology and microstructure of the samples were examined using field-emission scanning electron microscopy (FESEM, JEOL JSM-7900F, Tokyo, Japan) and transmission electron microscopy (TEM, JEOL JEM-1400F, Tokyo, Japan). Functional groups were identified by Fourier transform infrared spectroscopy (FT-IR, Bruker Vertex 70, Billerica, MA, USA), and the thermal stability of the materials was evaluated via thermogravimetric analysis (TGA, Netzsch TG209F1, Selb, Germany) under a nitrogen atmosphere. Phase composition was analyzed using X-ray diffraction (XRD, X’Pert Powder, Almelo, The Netherlands,), while surface chemical composition and bonding states were characterized by X-ray photoelectron spectroscopy (XPS, Thermo Fisher Scientific ESCALAB XI, Waltham, MA, USA). The compressive stress–strain behavior of the fibers was assessed using a universal testing machine (Instron-68SC-1, USA, Norwood, MA, USA). The real-time electrical signals and electrical characteristics of the piezoresistive sensor were recorded using an electrochemical workstation (Chenhua CHI760E, Shanghai, China). For wireless signal transmission, a commercial Bluetooth module (ESP8266, Espressif Systems, Shanghai, China) was employed in wearable sensor applications.

### 2.4. Preparation of TPU/CS/Ag Elastic Substrate

Thermoplastic polyurethane (TPU, 22 wt%) and chitosan (CS, 1 wt%) were dissolved in a mixed solvent of DMF and THF (mass ratio 1:1). The solution was stirred at 60 °C for 12 h until fully homogenized. It was then loaded into a 10 mL medical syringe fitted with a 21G stainless steel needle for electrospinning. During electrospinning, the flow rate was set at 0.5 mL/h, with an applied voltage of 12.5 kV at the needle tip. The distance between the needle and the rotating drum collector was maintained at 18 cm. Temperature and relative humidity were continuously monitored and controlled at 30 ± 2 °C and 50% RH, respectively. The resulting TPU/CS fibrous membrane was cut into 2 cm × 2 cm pieces, and conductive silver paste was uniformly brushed onto the membrane surface to form electrodes with a size of 1 cm × 2 cm.

### 2.5. Assembly of the PPDP-Based Flexible Sensor

The PPDP fibrous membrane was cut into 1 cm × 1 cm squares and laminated directly onto the flexible silver electrode. An adhesive glue (3M Vetbond 1469C) was used to fix and encapsulate the sensor structure, ensuring conformal contact between the PPDP membrane and the silver-coated electrode. Finally, two copper wires were connected to both ends of the Ag electrode using conductive silver paste to establish external electrical connections for signal acquisition.

### 2.6. Electromechanical Characterization

The electromechanical performance of the piezoresistive sensors was evaluated using a universal testing machine (Instron 68SC-1, Norwood, MA, USA) in conjunction with an electrochemical workstation (CHI760E, Chenhua, Shanghai, China). A 50 N load cell was employed for low-pressure measurements, while a 500 N load cell was used for high-pressure testing. For static sensing performance, the electrical signals were collected in real time via a computer connected to the electrochemical workstation during the application of constant pressure. Under dynamic loading, continuous electrical responses were recorded simultaneously. Unless otherwise specified, the average loading rate was set to 10 mm/min.

### 2.7. Practical Application of the PPDP Sensor on Human Subjects

The study only involved non-invasive surface attachment of flexible sensors for human motion detection and physiological signal monitoring. No invasive operations were performed on participants, and the research carried no potential risks to subjects. In accordance with the Measures for the Ethical Review of Research Involving Humans in Life Sciences and Medicine (Guowei Kejiao [2023] No. 4, Article 32, https://www.nhc.gov.cn/wjw/c100375/202302/902b4a1dc3af4aba862a6387e6e376dc.shtml, accessed on 29 June 2026) jointly issued by relevant national authorities of China, anonymized research that causes no harm to participants and does not involve sensitive personal information or commercial interests is exempted from mandatory institutional ethical review. Informed consent was obtained from all participants prior to their involvement, and all acquired data were fully anonymized upon collection.

## 3. Results and Discussion

### 3.1. Preparation and Characterization of PPDP Piezoresistive Sensor

**Figure 2:**
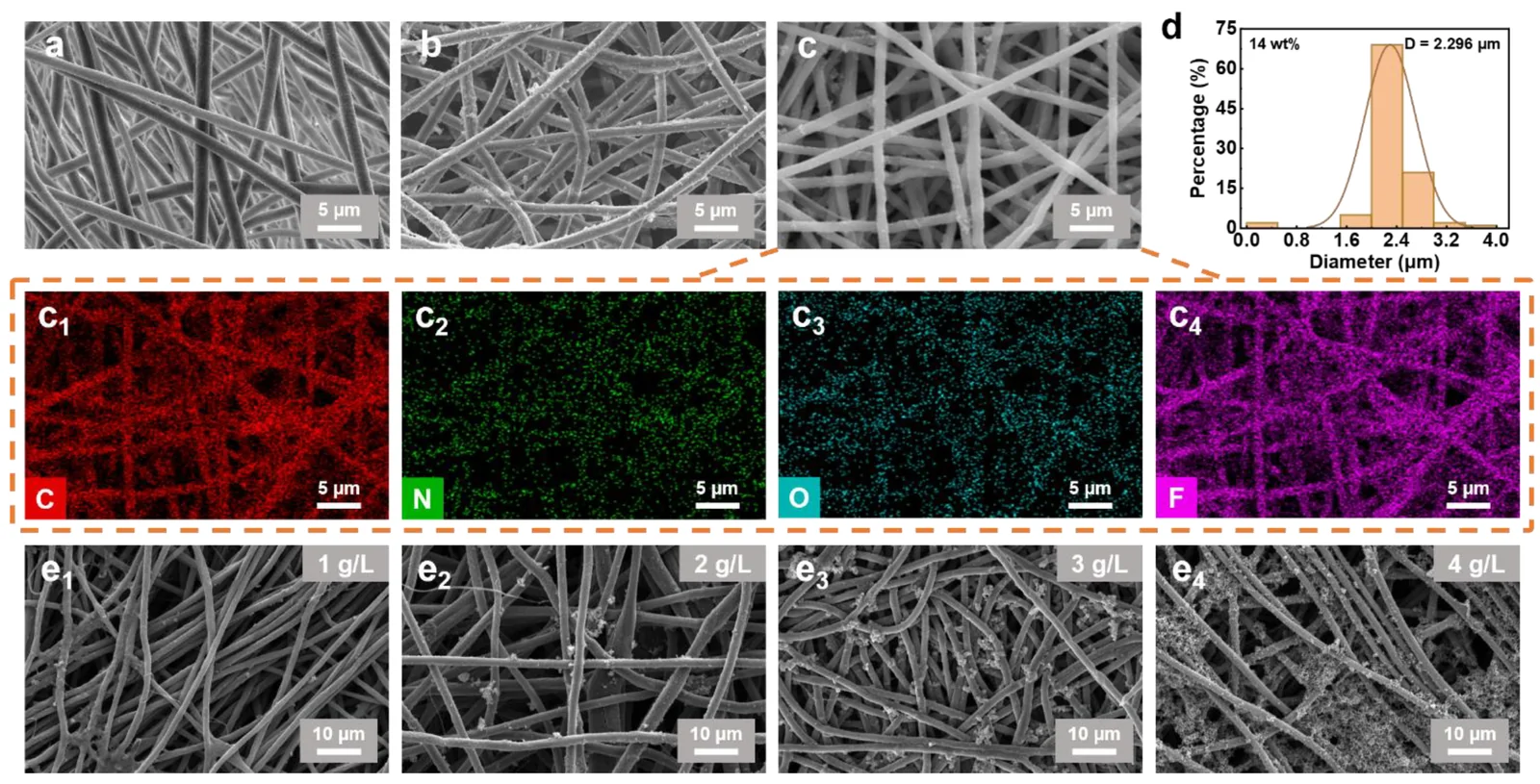
(**a**) SEM image of PP. (**b**) SEM image of PPD. (**c**) SEM image and EDS elemental mapping of PPDP, where (**c_1_**) corresponds to C, (**c_2_**) to N, (**c_3_**) to O, and (**c_4_**) to F. (**d**) Diameter distribution diagrams of 14 wt% PP. (**e**) SEM images of PPDP at different Py concentrations: (**e_1_**) = 1 g/L, (**e_2_**) = 2 g/L, (**e_3_**) = 3 g/L, and (**e_4_**) = 4 g/L.

The PPDP membrane with a fibrous structure was fabricated through a straightforward electrospinning and in situ polymerization method. Previous studies have shown that the fiber diameter tends to increase with the rising solid content of the electrospinning solution. As shown in Figure 2a,d and Figure S1, the average fiber diameter gradually increased from 0.879 μm at 10 wt% to 3.287 μm at 16 wt% as the PVDF/PAN concentration increased. The diameter distribution also became more uniform with higher concentrations, and the composited membrane electrospun from the 14 wt% solution exhibited the best uniformity. Therefore, this concentration was selected for subsequent experiments. As shown in Figure 2b, in situ polymerization of PDA on the electrospun PVDF/PAN nanofibers introduced numerous polar groups on the fiber surface, which is believed to facilitate the deposition of PPy nanoparticles on the PPD membrane surface [32]. PPy was subsequently polymerized in situ as the conductive component. SEM images and EDS elemental mapping (Figure 2c) confirmed the successful and uniform growth of PPy on the fibers. The detected nitrogen originated from PAN, PPy, and PDA; oxygen from PDA; and fluorine from PVDF.

To optimize the polymerization conditions for PPy, the effect of varying the molar ratio between pyrrole and the oxidant was investigated. As shown in Figure S2, increasing the proportion of FeCl_3_·6H_2_O led to a significant increase in PPy particle density, due to the enhanced polymerization rate. Insufficient oxidant resulted in incomplete polymerization, while excessive oxidant caused over-rapid growth and particle aggregation, which may disrupt the uniformity of conductive pathways. At a Py-to-oxidant ratio of 1:4, the fiber surface was fully covered by PPy, forming a continuous conductive network, which may adversely affect sensing performance [33]. Figure 2e, Figures S3 and S4 display the polymerization states at different Py concentrations. As the Py concentration increased, the number of PPy particles also increased. However, at higher concentrations, particle aggregation was observed, attributed to accelerated polymerization that hinders uniform deposition. The resulting PPDP membrane exhibited good flexibility in Figure S5, with no observable damage or structural change under bending and twisting.

**Figure 3:**
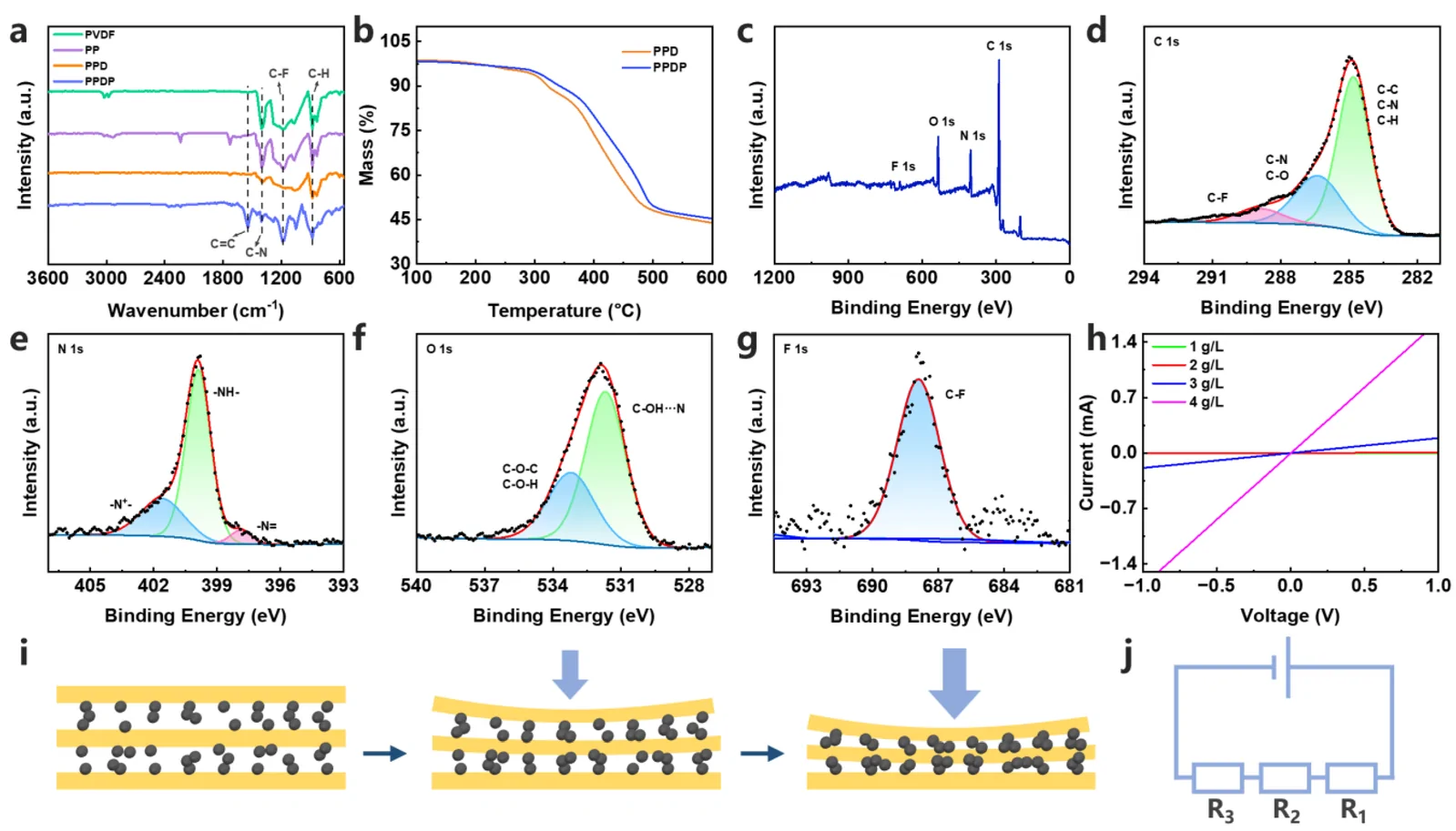
(**a**) FT-IR spectra of PVDF, PP, PPD and PPDP. (**b**) TGA spectra of PPD and PPDP. (**c**) XPS full spectrum curve of PPDP. (**d**–**g**) C 1s, N 1s, O 1s, and F 1s XPS high-resolution narrow spectrum of PPDP, respectively. The black dots represent the original experimental XPS data; the red solid line represents the cumulative fitting curve; the colored filled curves represent the individual deconvoluted peaks corresponding to specific chemical bonds/functional groups; and the bottom baseline represents the background signal (**h**) I−V curves of the PPDP fibrous membrane prepared with different pyrrole concentrations. (**i**) Schematic diagram of the sensing mechanism. (**j**) PPDP piezoresistive sensor equivalent circuit diagram.

Further characterizations were conducted to investigate the structural and compositional differences between the PPD and PPDP fibrous membranes. As shown in Figure 3a, a distinct peak at 1543 cm^−1^ corresponds to the characteristic symmetric stretching vibration of the C=C bonds in PPy [34]. The peak at 1635 cm^−1^ corresponds to the C=N covalent bond formed between PDA and PPy. Peaks at 1404 cm^−1^ and 1180 cm^−1^ are associated with the stretching vibrations of C–N and C–F bonds, respectively [32,35], while the peak at 879 cm^−1^ is attributed to the in-plane bending vibration of C–H bonds [36]. FT-IR analysis confirms the successful polymerization of PPy and its interaction with PDA through both hydrogen bonding and covalent bonding. The thermal stability of PPD and PPDP was assessed by thermogravimetric analysis (TGA), as shown in Figure 3b. Both membranes exhibit minimal mass loss below 100 °C, indicating good thermal stability in this range. As the temperature increases, the phenolic hydroxyl and amine groups in PPD begin to decompose, leading to a more pronounced mass loss. In contrast, the PPDP membrane undergoes a slower degradation process due to the superior thermal stability of the PPy backbone. This result confirms the successful incorporation of PPy into the fibrous structure. XRD analysis in Figure S6 reveals two distinct diffraction peaks at 18.3° and 20.2°, corresponding to the α- and β-phases of PVDF, respectively [37]. X-ray photoelectron spectroscopy (XPS) was also employed to analyze the surface composition of PPD and PPDP, as shown in Figure S7a and Figure 2c. Compared to PPD, the PPDP membrane shows a lower fluorine content and a higher carbon content, attributed to the presence of carbon-rich PPy particles formed via in situ polymerization on the fiber surface. High-resolution XPS spectra (Figure S7b–e and Figure 3d–g) further elucidate the bonding interactions between the PPy particles and the fiber matrix. In the C 1s spectrum (Figure 3d), the binding energy at 286.37 eV corresponds to C=N and C=O bonds, primarily originating from PPy and PDA. In comparison, the PPD membrane (Figure S7b) exhibits only C–O bonds from PDA. In the N 1s spectrum (Figure 3e), binding energies at 397.86 eV, 399.88 eV, and 401.61 eV are assigned to =N–, –NH–, and –N^+^– groups, respectively. In contrast, the N 1s spectrum of PPD (Figure S7c) exhibits only a single peak at 399.73 eV, which is attributed to the –NH– groups from PDA. The O 1s spectrum (Figure 3f) displays peaks at 531.69 eV and 533.20 eV, corresponding to C–OH⋯N, C–O–C, and C–O–H groups. These signals differ significantly from those of PPD (Figure S7c,d), confirming that the PPy particles are strongly anchored to PDA via hydrogen bonding and covalent interactions.

To investigate the effects of monomer-to-oxidant ratio and monomer concentration on the PPy polymerization process, electrochemical measurements were carried out. As shown in Figure 3h and Figure S8, increasing the FeCl_3_·6H_2_O content or the pyrrole concentration accelerates the polymerization rate, resulting in reduced resistance and improved conductivity of the membrane. However, excessively fast polymerization can lead to PPy aggregation, hindering uniform distribution and affecting the formation of effective conductive pathways.

A schematic illustration of the working mechanism of the PPDP-based piezoresistive sensor is shown in Figure 3i. In the absence of pressure, limited conductive pathways exist, resulting in weak current signals. When a small pressure is applied, more conductive pathways form, leading to stronger current output. Under higher pressure, extensive conductive networks are established, further enhancing the current signal. This pressure-dependent conductivity is attributed to the PPy particles uniformly distributed on the fibers, forming a three-dimensional conductive network. The sensor translates changes in contact area into measurable current outputs, enabling accurate pressure monitoring. Figure 3j illustrates the resistive components of the sensor, including fiber resistance, inter-fiber contact resistance, and electrode–fiber contact resistance.

### 3.2. Preparation and Characterization of TC/PPDP Piezoresistive Sensor

**Figure 4:**
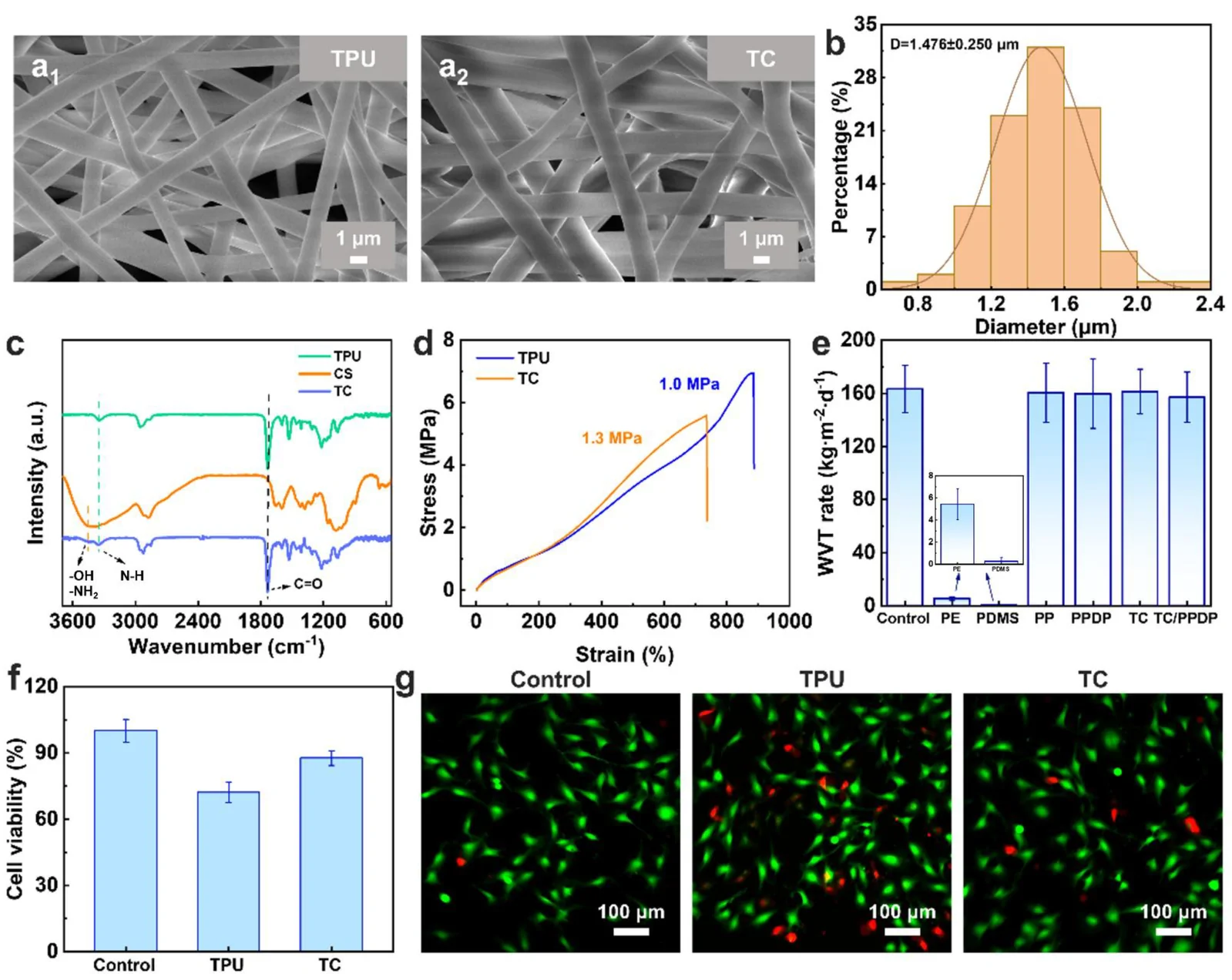
(**a_1_**,**a_2_**) SEM images of TPU and TC. (**b**) Diameter distributions of TC fibers. (**c**) FTIR spectra of different compositions. (**d**) Stress–strain curves of TPU and TC membranes. (**e**) Comparison of water vapor transmission rates. (**f**) Cell viability of TPU and TC fibrous membranes. (**g**) Live/Dead cell staining images of TPU and TC fibrous membranes.

To fabricate the protective layer of the PPDP sensor, elastic TPU/CS (TC) fibrous membranes were prepared via electrospinning. TPU fibrous membranes with different polymer concentrations were first fabricated, and their SEM images are shown in Figure 4a_1_, Figures S9 and S10. Pronounced beaded and spindle-like morphologies were observed at TPU concentrations of 18 wt% and 20 wt%, respectively. As the TPU concentration increased, the fiber diameter gradually increased, and continuous fibers were obtained at a concentration of 22 wt%. Subsequently, CS was incorporated into the TPU spinning solution for blend electrospinning. The SEM image of the resulting TC membrane (Figure 4a_2_) shows continuous fibers with a relatively uniform diameter distribution. Statistical analysis revealed that the average fiber diameter increased from 1.185 μm for TPU fibers to 1.476 μm after the addition of CS (Figure 4b and Figure S9). The FTIR spectra of TPU, CS, and TC membranes are presented in Figure 4c. The broad absorption band centered at 3455 cm^−1^ is attributed to the –OH and –NH_2_ groups of CS, while the characteristic peak at 3346 cm^−1^ corresponds to the stretching vibration of the –NH bond in TPU. The characteristic C=O stretching vibration of TPU appears at 1736 cm^−1^, whereas the corresponding peak in the TC membrane shifts to 1730 cm^−1^. This red shift is likely associated with the involvement of carbonyl groups in hydrogen-bond formation, indicating intermolecular interactions between TPU and CS within the blended system. Optical photographs of the TC membrane before and after stretching are shown in Figure S12. The membrane exhibited typical elastic behavior. After being subjected to tensile deformation, the TC membrane largely recovered its original appearance without obvious yielding, demonstrating good shape recovery capability, which is beneficial for maintaining stable signal output during sensor operation. Figure 4d compares the stress–strain curves of TPU and TC membranes. The TPU membrane exhibited an elongation at break of 885% and a Young’s modulus of 1.0 MPa. After the incorporation of CS, the elongation at break decreased to 734%, while the modulus increased to 1.3 MPa. To evaluate the feasibility of long-term wearable applications, the breathability and biocompatibility of the TC/PPDP sensor were investigated. As shown in Figure 4e, both PPDP and TC membranes exhibited high water vapor transmission rates (WVTRs). Although the WVTR slightly decreased after sensor assembly, the TC/PPDP membrane still maintained a value of 156.96 kg·m^−2^·d^−1^. Such high vapor permeability is favorable for the rapid evaporation of perspiration during prolonged wear. The biocompatibility of the TC membrane was assessed through in vitro cell culture experiments. Cell viability was evaluated using the MTT assay, and the results are presented in Figure 4f. Compared with the control group and the TPU membrane, the TC-1 membrane exhibited the highest cell viability, exceeding 87.6%. Live/dead cell staining was further performed using PI staining to evaluate cytocompatibility (Figure 4g). Stronger green fluorescence was observed in both the control and TC groups, whereas noticeable red fluorescence appeared in the TPU group. These results indicate that cells remained viable in the control and TC groups, while partial cell death occurred in the TPU group. Overall, the findings demonstrate that the TC membrane possesses favorable biocompatibility.

### 3.3. Pressure-Sensing Properties of the PPDP-Based Sensor

**Figure 5:**
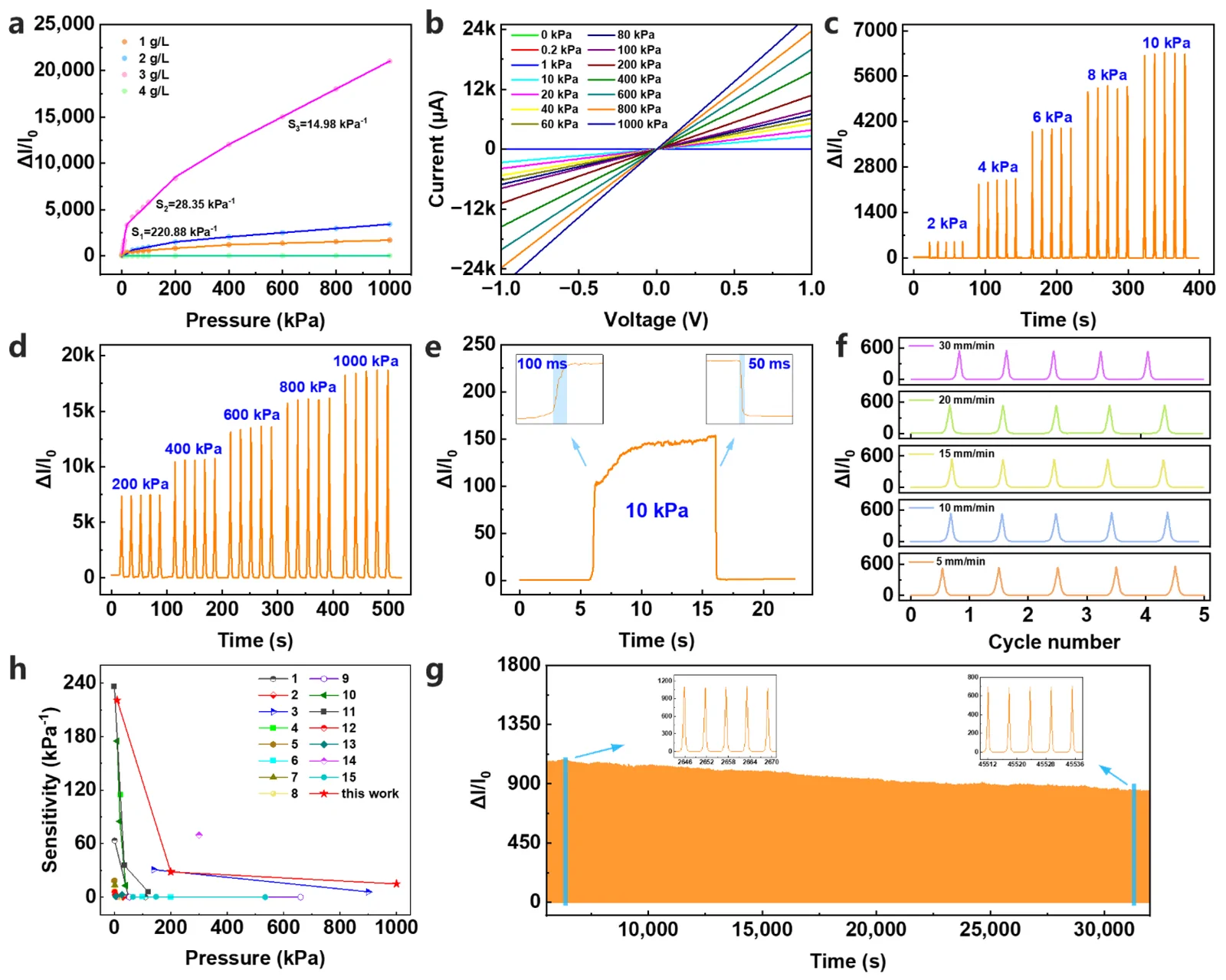
(**a**) Sensitivity of PPDP-based sensors under 0-1 MPa. (**b**) CV curves of PPDP sensor with different applied pressures. (**c**) I-T curves of PPDP under low applied pressure changes. (**d**) I-T curves of PPDP under large applied pressure changes. (**e**) Response and recovery time of the prepared sensors under 10 kPa at 5 mm/s. (**f**) I-T curves of PPDP with different running speeds. (**g**) Excellent repeatability after continuous 5000 loading–unloading cycles under 50 kPa. (**h**) Performance comparison between our work and the reported piezoresistive pressure sensors. (1) [41], (2) [42], (3) [43], (4) [44], (5) [45], (6) [46], (7) [47], (8) [48], (9) [49], (10) [50], (11) [51], (12) [52], (13) [53], (14) [54], (15) [55].

To achieve optimal device performance, PPDP-based piezoresistive sensors were fabricated using different pyrrole concentrations, and their sensing properties were systematically evaluated. Sensitivity, as a key parameter for assessing sensor performance, is typically defined by Equation (1) [38,39]:

(1) $$S=\frac{\Delta I/I_{0}}{\Delta P}=\frac{I_{0}-I}{I_{0}\Delta P}$$

where ΔI represents the current change upon applied pressure, I is the current under applied pressure, I_0_ is the baseline current under zero pressure, and ΔP is the applied pressure. Figure 5a presents the sensitivity of PPDP sensors fabricated with different Py concentrations. The detailed sensitivity–pressure curves corresponding to 1, 2, 3, and 4 g/L Py concentrations are shown in Figure S11. The results indicate that the sensors exhibit three distinguishable linear regions over the full pressure range of 0–1 MPa, with sensitivity decreasing as pressure increases. Notably, sensitivity first increases and then decreases with increasing Py concentration, reaching a maximum at 3 g/L. In the low-pressure regime (<10 kPa), the sensor achieves a high sensitivity of 220.88 kPa^−1^, while in the medium- (10–200 kPa) and high-pressure ranges (200–1000 kPa), the sensitivities are 28.35 kPa^−1^ and 14.98 kPa^−1^, respectively. This trend can be attributed to the morphology of PPy particles formed under different Py concentrations. At 3 g/L, PPy particles are uniformly and densely distributed on the fiber surface, enabling the formation of efficient conductive pathways even under slight pressure. At lower concentrations, the polymerization rate is slower, resulting in sparsely distributed PPy particles that limit the formation of conductive networks. In contrast, higher Py concentrations promote rapid polymerization, leading to particle aggregation and non-uniform coverage. In some cases, excessive PPy growth may fully coat the fiber surface, limiting the responsiveness to additional pressure. Additionally, the multilayered structure of the sensing layer significantly influences the sensor’s performance (Figure S13). An increased number of layers enhances the quantity of inter-fiber contact points, thereby improving the sensitivity. However, this comes at the cost of increased hysteresis, as previously reported [40].

Figure 5b shows the cyclic voltammetry (CV) curves of the PPDP sensor under varying pressures. The current increases linearly with pressure, confirming the device’s reliable sensing behavior across different environments. As shown in Figure 5c,d, the sensor provides consistent current responses in both low- (2–10 kPa) and high-pressure (200–1000 kPa) ranges, with signal intensity increasing proportionally with pressure. The response and recovery times were evaluated under 10 kPa pressure using a loading speed of 5 mm/s. As shown in Figure 5e and Figure S14, the response and recovery times were approximately 100 ms and 50 ms, respectively, with minimal variation under different pressure levels. Furthermore, Figure 5f and Figure S15 demonstrate consistent output currents under different compression speeds at the same pressure, indicating excellent dynamic stability. To assess durability, fatigue testing was performed at a loading speed of 20 mm/min for 5000 cycles. As shown in Figure 5g, the signal amplitude showed only minor attenuation after repeated cycles, confirming the PPDP sensor’s outstanding stability and long service life.

By comparing the performance parameters of various piezoresistive sensors listed in the table, the PVDF/PAN/PDA/PPy (PPDP) sensor demonstrates significant comprehensive advantages. In the low-pressure region (<10 kPa), it achieves a high sensitivity of 220.88 kPa^−1^, outperforming most compared devices; in the medium-pressure and high-pressure regions, it maintains sensitivities of 28.35 kPa^−1^ and 14.98 kPa^−1^, respectively, enabling an ultra-wide detection range of 0–1000 kPa, far exceeding the sub-hundred-kPa working ranges of most reported devices. Moreover, its response/recovery times are only 100/50 ms, and its cycling stability is excellent, both surpassing comparable works (Table 1). Figure 5h compares the performance of the PPDP sensor with previously reported piezoresistive sensors. Although the sensitivity and pressure range are not the highest reported to date, the PPDP sensor achieves a favorable balance between high sensitivity and a wide working range, making it suitable for diverse pressure monitoring applications and demonstrating strong potential for practical use.

### 3.4. Practical Application of the PPDP Sensor

**Figure 6:**
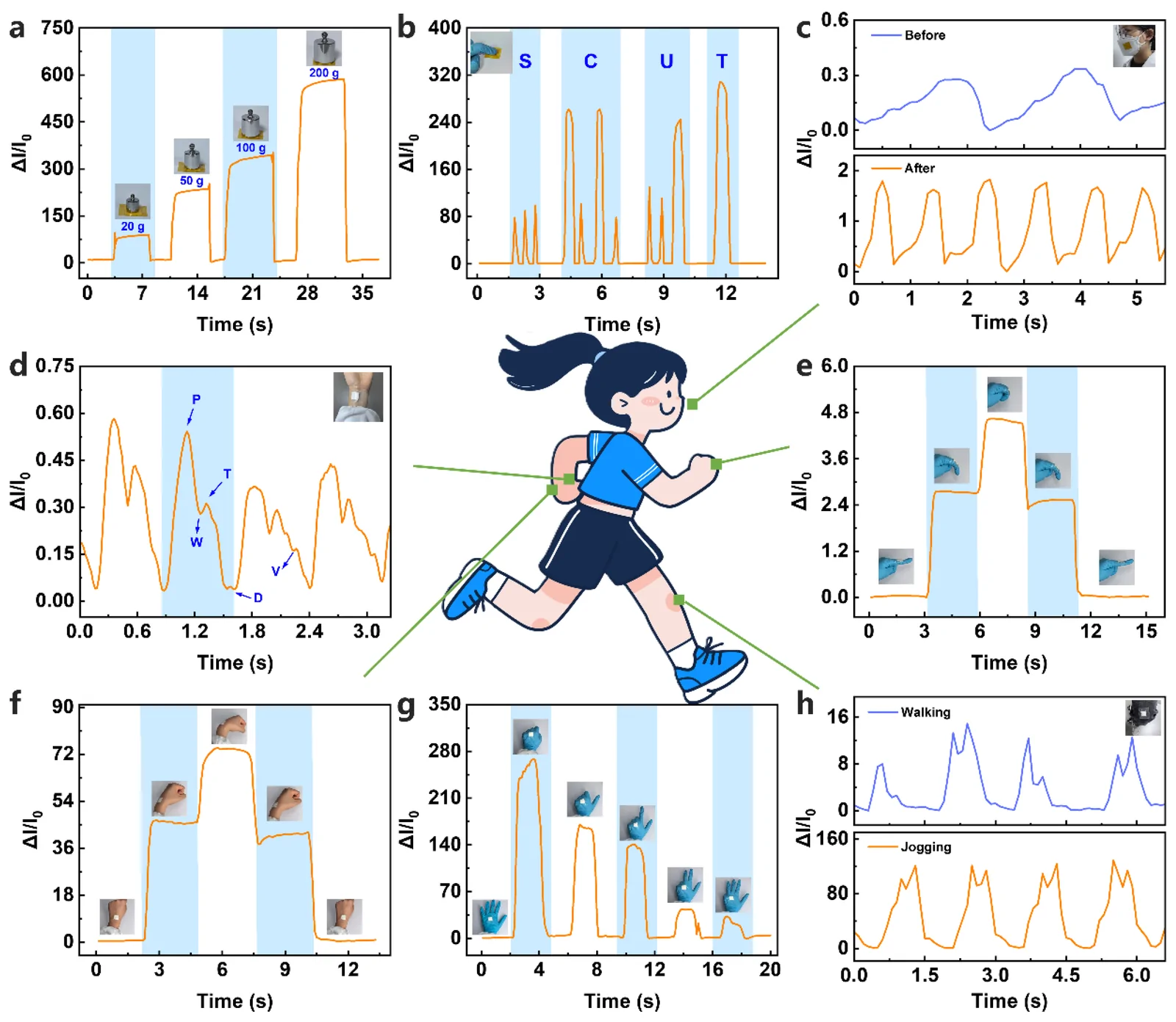
Applications of the PPDP piezoresistive sensor for human motion detection. (**a**) Output signals under different weights. (**b**) Sending international Morse code (SCUT) by taps and long presses on the test platform. (**c**) Respiration through masks before and after exercises. (**d**) Radial artery pulse waveform in volunteers at rest. (**e**) Different fingers’ bending signal. (**f**) Wrist joint. (**g**) Finger knuckle movements. (**h**) Knee flexion.

Based on the above experiments, the fabricated PPDP sensor exhibits high sensitivity, a broad pressure detection range, and rapid response/recovery time, making it a promising candidate for wearable piezoresistive devices capable of real-time human motion monitoring and recognition. To demonstrate its practical potential, a series of application tests were conducted. As shown in Figure 6a, placing weights of varying masses on the PPDP sensor resulted in proportional current changes, while heavier weights generated larger current responses. This feature enables the estimation of applied pressure and could be used, for instance, to detect falls in elderly individuals. Furthermore, the sensor was tapped according to Morse code patterns to differentiate between short and long signals (Figure 6b), offering a potential communication aid for patients with Parkinson’s disease who struggle with verbal expression. The PPDP sensor also demonstrates excellent performance in physiological monitoring. When attached to an N95 face mask using Kapton tape, it effectively detected respiratory frequency during breathing cycles (Figure 5c). Additionally, when placed on the wrist, the sensor successfully captured pulse waveforms (Figure 5d). Not only were key features such as the percussion wave (P-wave), tidal wave (T-wave), and diastolic wave (D-wave) clearly distinguishable, but the sensor also identified secondary features including the reflected notch (W) and dicrotic notch (V), indicating its potential for cardiovascular health monitoring [56]. In motion detection applications, the PPDP sensor was applied to monitor finger and wrist joint bending (Figure 6e,f), which can be used for diagnosing tenosynovitis or recognizing specific hand gestures. This functionality also supports sign language recognition for hearing-impaired individuals (Figure 6g). Furthermore, the sensor was used to track knee motion during walking and running (Figure 6h), indicating potential for gait analysis and rehabilitation monitoring. These results confirm that the PPDP sensor delivers excellent real-time responsiveness and versatility in detecting both subtle physiological signals and complex limb movements. With its comprehensive sensing capabilities and flexibility, the PPDP sensor holds substantial promise for integration into next-generation wearable health monitoring systems and intelligent human–machine interfaces.

## 4. Conclusions

In summary, this study presents a simple and effective strategy for fabricating a high-performance flexible piezoresistive sensor with both high sensitivity and a broad pressure range. A high-surface-area fibrous structure was constructed via electrospinning, followed by surface modification with PDA and in situ polymerization of PPy. The PPy was anchored onto the PDA-modified fibers through both hydrogen bonding and covalent bonding. By precisely tuning the PPy growth conditions, a three-dimensional conductive network with excellent sensing performance was achieved. In addition, an elastic and biocompatible substrate was also fabricated via electrospinning to encapsulate the sensing layer, endowing the PPDP sensor with superior mechanical stability and skin conformability. The resulting sensor exhibited an ultrahigh sensitivity of up to 220.88 kPa^−1^ in the low-pressure range (0–10 kPa) and maintained stable performance across a wide pressure range up to 1 MPa. Moreover, the device demonstrated excellent durability, withstanding over 5000 loading/unloading cycles at 50 kPa without significant signal degradation. These features enable real-time monitoring and recognition of various human physiological signals, highlighting the great potential of the PPDP sensor in next-generation artificial intelligence systems and future wearable health monitoring technologies.

## Figures and Tables

**Table 1:** Comparison of the performance between the proposed piezoresistive pressure sensor and existing research.

Pressure Sensor	Sensitivity (kPa^−1^)				Response	Pressure Range	Ref.
	Part 1	Part 2	Part 3	Part 4	Time		
TPU/PANI (EFM-OMA)	63.41 (<1 kPa)	0.64 (1–50 kPa)	0.08 (50–110 kPa)	\	62 ms	0–110 kPa	[41]
CM/CA/TPU	2.1 (<5 kPa)	1.2 (5–10 kPa)	0.4 (10–30 kPa)	\	<30 ms	0–25 kPa	[42]
loofah/MWCNTs	30.8 (<140 kPa)	6.0 (140–900 kPa)	\	\	64 ms	0–900 kPa	[43]
CA/SA/MXene	114.55 (<21.78 kPa)	\	\	\	150 ms	0–21.78 kPa	[44]
graphene fiber aerogel (GFA)	18.55 (<0.38 kPa)	2.73 (0.38–4 kPa)	0.42 (4–7 kPa)	\	2 ms	0–7.5 kPa	[45]
LIG/PVP	0.338 (1–100 kPa)	0.015 (100–200 kPa)	\	\	31 ms	0–200 kPa	[46]
dextran@IL	13.7 (<2 kPa)	\	\	\	22 ms	0–2 kPa	[47]
Ti_3_C_2_/Na_2_S	7.8 (<1 kPa)	1.11 (1–5 kPa)	0.03 (5–20 kPa)	\	50 ms	0–20 kPa	[48]
SEBS-BTO-CNT	0.068 (<53 kPa)	0.013 (53–660 kPa)	\	\	25 ms	0–660 kPa	[49]
T-ZnOw/PDMS	175 (<10 kPa)	85 (10–20 kPa)	\	\	430 ms	0–40 kPa	[50]
MXene@ZnO/Mxene/PU	236.5 (<0.2 kPa)	35.8 (0.2–35 kPa)	6.1 (35–120 kPa)	1.2 (120–260 kPa)	100 ms	0–260 kPa	[51]
MWCNTS/cellulose	6.09 (<0.92 kPa)	2.88 (0.92–2.63 kPa)	1.35 (2.63–5.05 kPa)	0.51 (5.05–10 kPa)	<280 ms	0–10 kPa	[52]
L-Ti_3_C_2_/PI	0.83 (<5.3 kPa)	2.65 (5.3–27.1 kPa)	\	\	\	0–27.1 kPa	[53]
TPU@MWCNTs	69.8 (<300 kPa)	\	\	\	1 ms	0–300 kPa	[54]
MXene/PDMS	0.255 (6.6–66 kPa)	0.432 (66–148 kPa)	0.066 (148–535 kPa)	\	57 ms	0.033–535 kPa	[55]
PVDF/PAN/PDA/PPy	220.88 (<10 kPa)	28.35 (10–200 kPa)	14.98 (200–1000 kPa)	\	100 ms	0–1 MPa	This work

## Data Availability

The original contributions pre-sented in this study are included in the article/Supplementary Material. Further inquiries can be directed to the corresponding authors.

## Supplementary Materials

The following supporting information can be downloaded at: https://www.mdpi.com/article/10.3390/polym18161937/s1, Figure S1: (a) SEM images of PP (scale bar: 1 μm). (b) SEM images of PP (scale bar: 10 μm). (c) Diameter distribution diagrams of PP; Figure S2: SEM images of different Py-to-oxidant ratios (a) Scale bar: 1 μm. (b) Scale bar: 10 μm; Figure S3: SEM images of different Py concentrations (scale bar: 1 μm); Figure S4: Diameter distribution of PPy nanoparticles in the SEM image of Figure 2e; Figure S5: Flexibility of PPDP film. (a) The distorting of PPDP film, (b and c) The bending of PPDP film; Figure S6: XRD spectrum of PVDF, PP, PPD and PPDP; Figure S7: (a) XPS full spectrum curve of PPD. XPS high resolution narrow-spectrum curve of (b) C, (c) N, (d) O, and (e) F in PPD fibers; Figure S8: I-V curves of PPDP membranes prepared with different ratios of oxidant to pyrrole; Figure S9: SEM image of TPU fibers at different mass concentrations; Figure S10: Diameter diagrams of 22wt% TPU; Figure S11: Optical images of TC membrane: (a) initial, (b) stretched, and (c) recovered; Figure S12: Sensitivity curves of PPDP at different concentrations of (a)1 g/L, (b) 2 g/L, (c) 3 g/L, (d) 4 g/L; Figure S13: Sensitivity curves of PPDP at different layers; Figure S14: Response and recovery times of the prepared sensors at 5 mm s-1 at different pressures under (a) 10 kPa, (b) 30 kPa, (c) 70 kPa, (d) 90 kPa; Figure S15: The I-T variation with different running speeds of the uniaxial motion controller.
