# Supplementary material for: High-Performance Flexible Piezoresistive Sensors Based on Covalently Anchored Polypyrrole Networks on Electrospun Fibrous Membranes
Source: Polymers (Basel). 2026 Aug 7;18(16):1937. doi: 10.3390/polym18161937 (PMC13516175; doi:10.3390/polym18161937)
Supplement: Supplementary file 1 [file polymers-18-01937-s001.zip › polymers-4438079-supplementary.pdf]

SUPPLEMENTARY INFORMATION of

High-Performance Flexible Piezoresistive Sensors Based on Covalently Anchored Polypyrrole Networks on Electrospun Fibrous Membranes

Zhifei Liang [1,†], Fangrong Tan [1,†], Xinyu Zeng [1], Xiao Su [1], Zhe Tang [1], Paul D. Topham [2], LinGe Wang [1,3,*] and Qianqian Yu [1,*]

[1] South China Advanced Institute for Soft Matter Science and Technology, School of Emergent Soft Matter, Guangdong Provincial Key Laboratory of Functional and Intelligent Hybrid Materials and Devices, South China University of Technology, Guangzhou 510640, China
[2] Aston Institute for Membrane Excellence, College of Engineering and Physical Sciences, Aston University, Birmingham B4 7ET, UK
[3] Guangdong Basic Research Center of Excellence for Energy and Information Polymer Materials, South China University of Technology, Guangzhou 510640, China
* Correspondence: lingewang@scut.edu.cn (L.W.); yuqianqian@scut.edu.cn (Q.Y.)
† These authors contributed equally to this work.

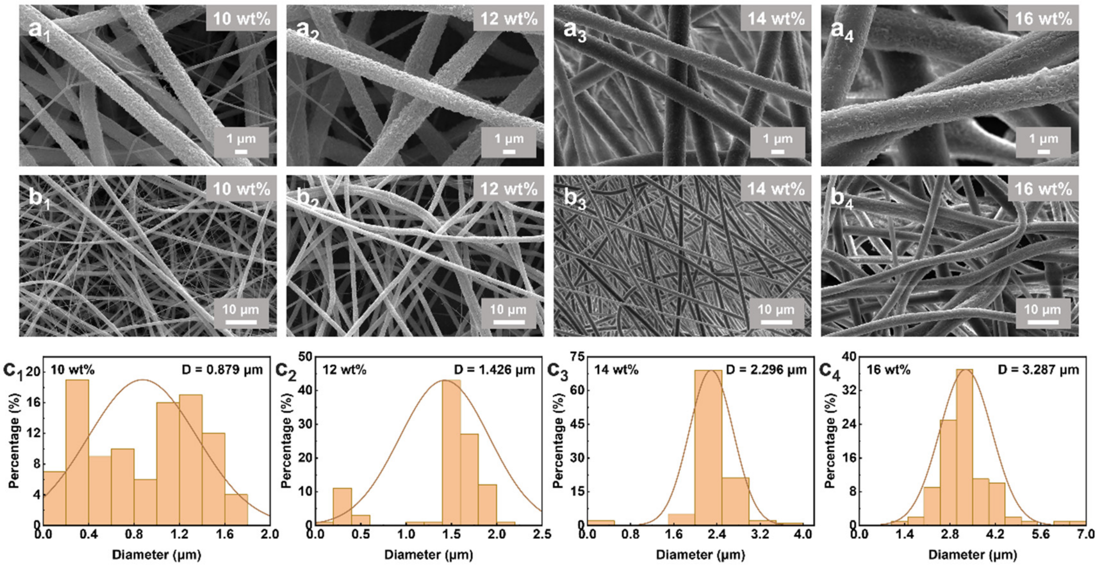

Figure S1. (a) SEM images of PP (scale bar: 1 μm). (b) SEM images of PP (scale bar: 10 μm). (c) Diameter distribution diagrams of PP.

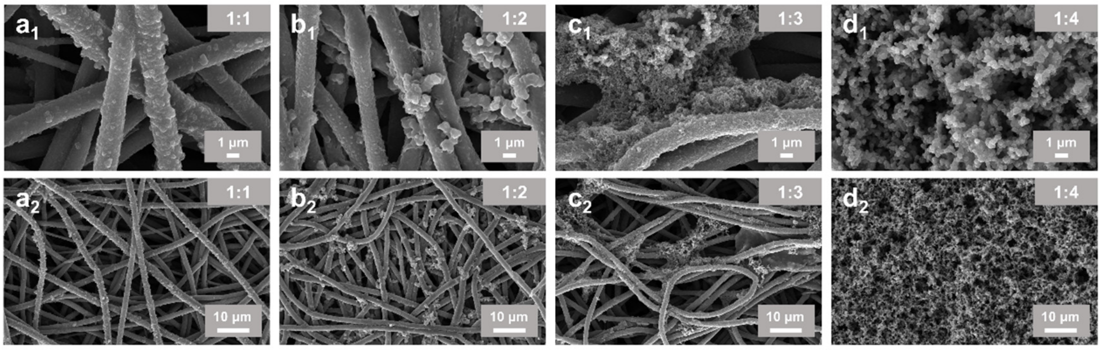

Figure S2. SEM images of different Py-to-oxidant ratios. (a) Scale bar: 1 μm. (b) Scale bar: 10 μm.

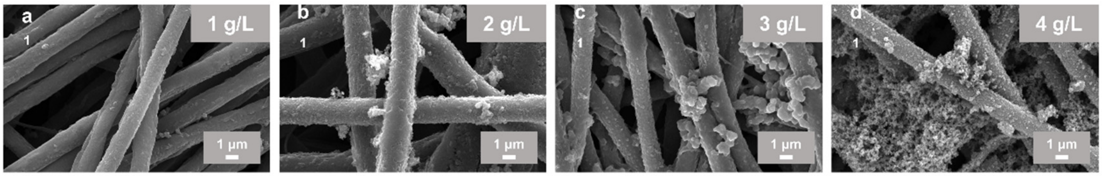

Figure S3. SEM images of different Py concentrations (scale bar: 1 μm).

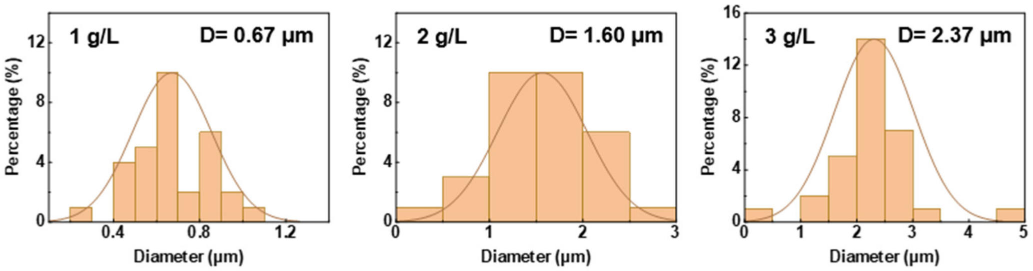

Figure S4. Diameter distribution of PPy nanoparticles in the SEM image of Figure 2e.

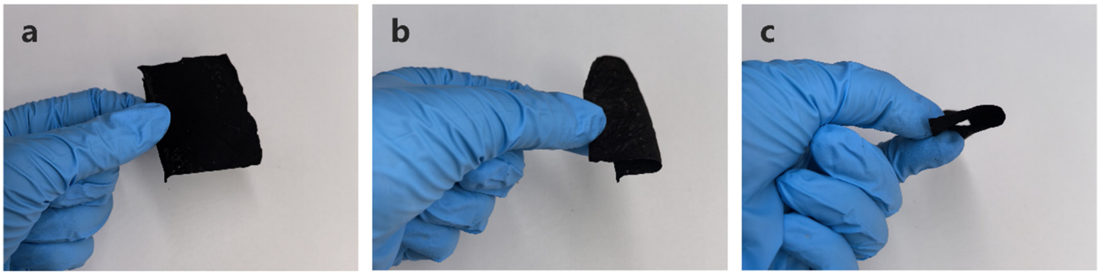

Figure S5. Flexibility of PPDP film. (a) The distorting of PPDP film; (b and c) the bending of PPDP film.

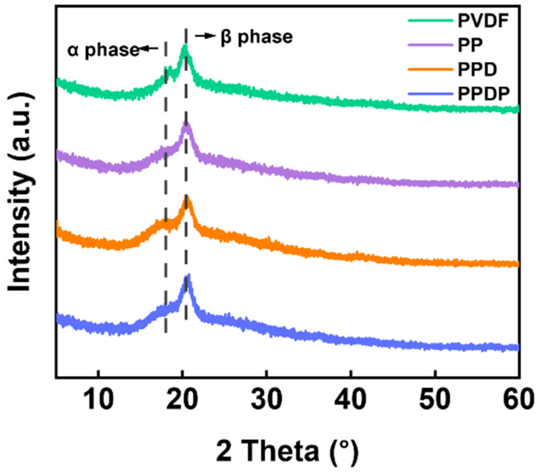

Figure S6. XRD spectrum of PVDF, PP, PPD and PPDP.

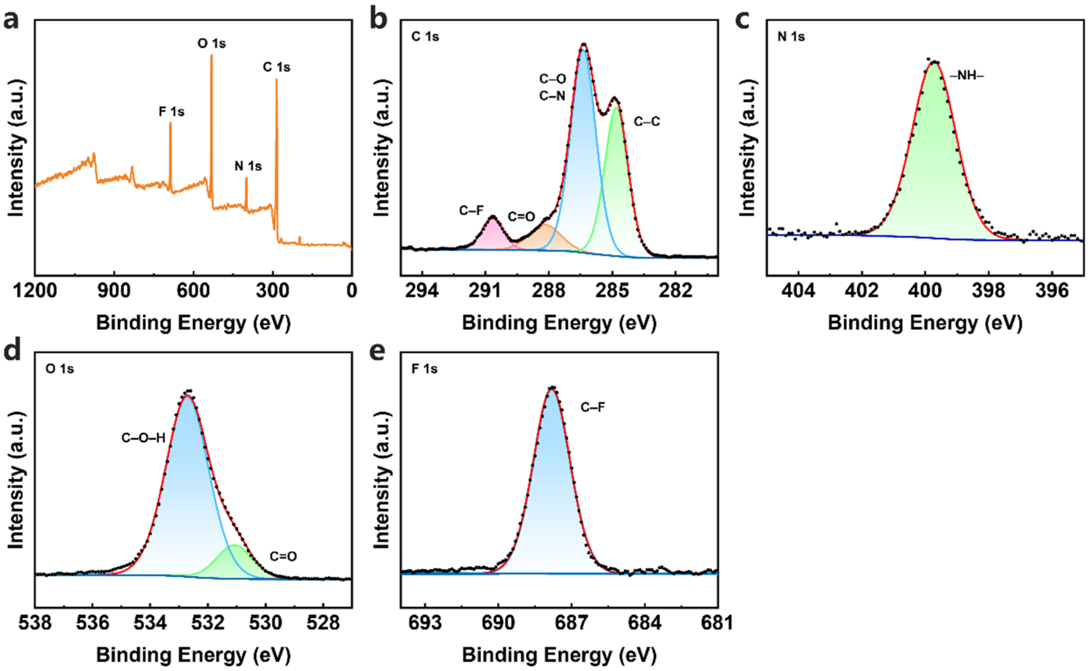

Figure S7. (a) XPS full spectrum curve of PPD. XPS high-resolution narrow-spectrum curve of (b) C, (c) N, (d) O, and (e) F in PPD fibers.

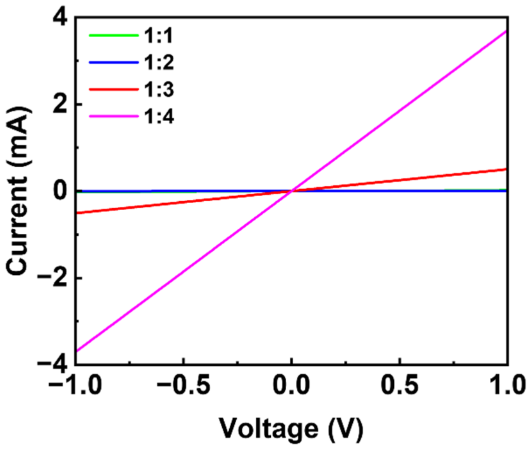

Figure S8. I-V curves of PPDP membranes prepared with different ratios of oxidant to pyrrole.

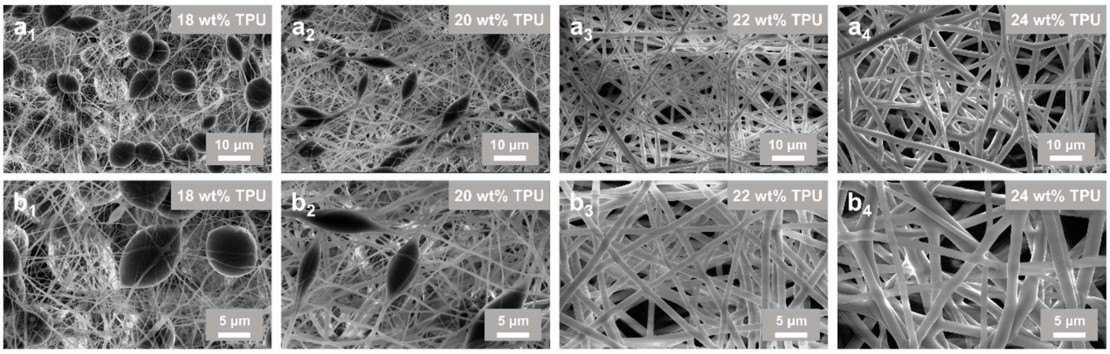

Figure S9. SEM image of TPU fibers at different mass concentrations.

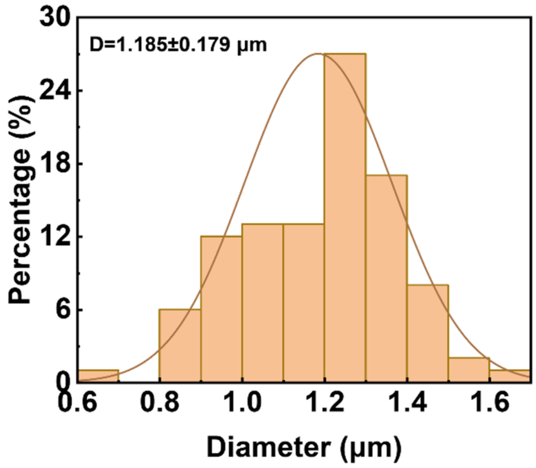

Figure S10. Diameter diagrams of 22wt% TPU.

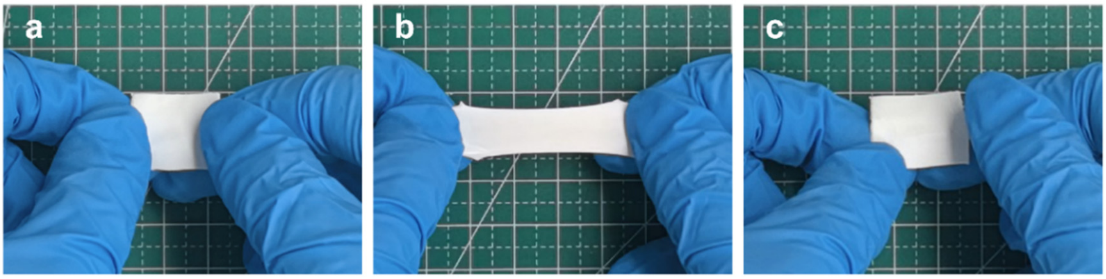

Figure S11. Optical images of TC membrane: (a) initial, (b) stretched, and (c) recovered.

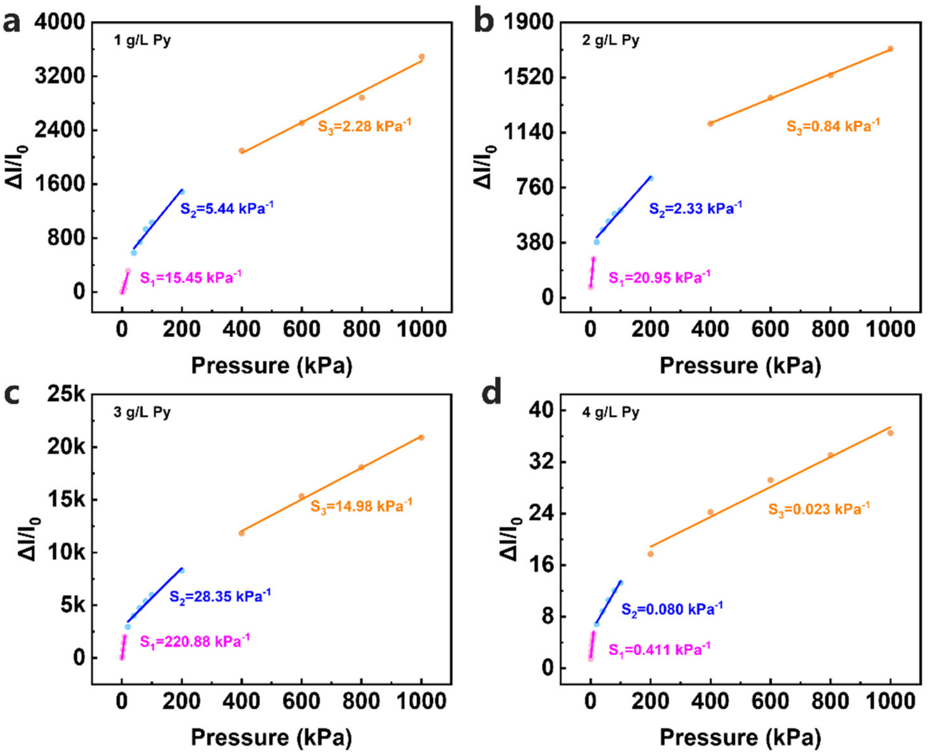

Figure S12. Sensitivity curves of PPDP at different concentrations of (a)1 g/L, (b) 2 g/L, (c) 3 g/L, (d) 4 g/L.

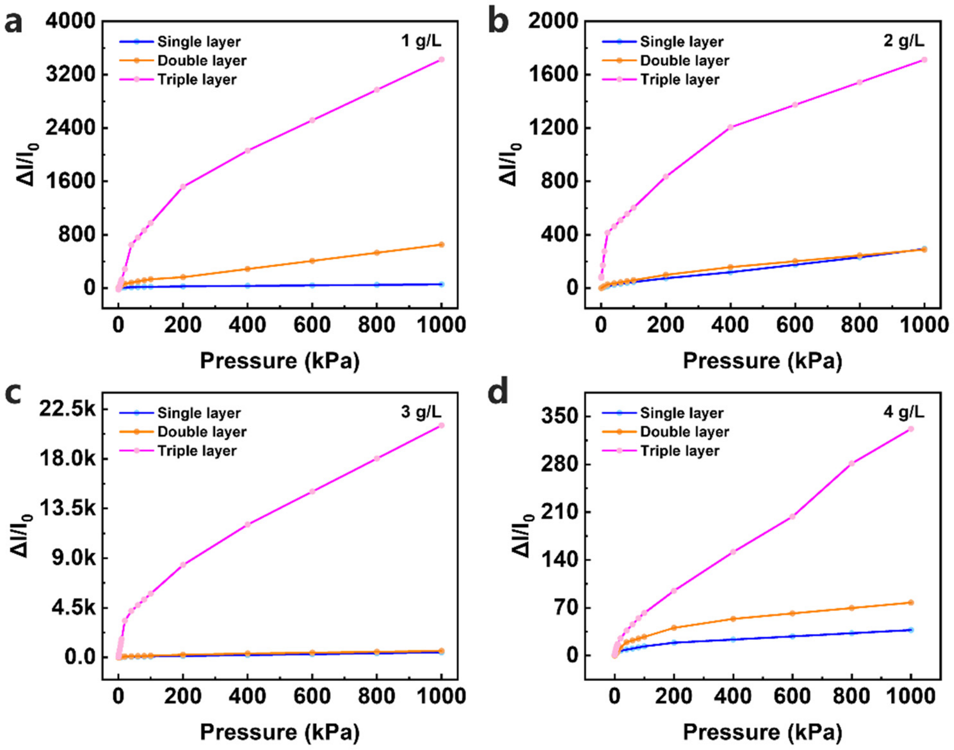

Figure S13. Sensitivity curves of PPDP at different layers.

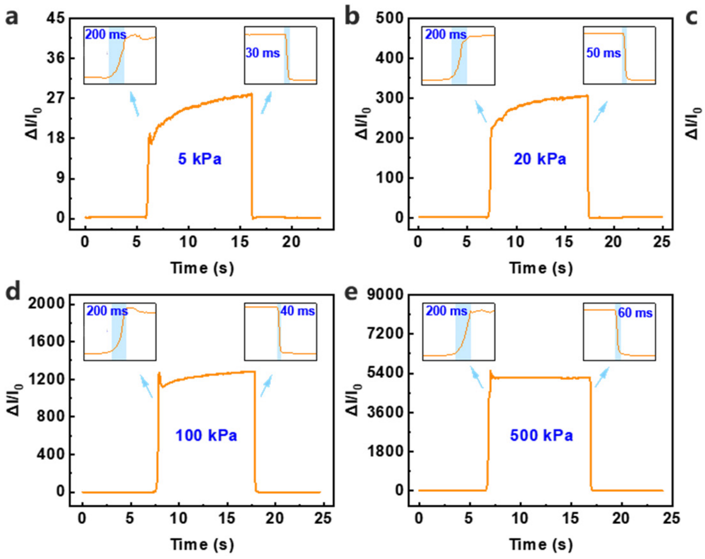

Figure S14. Response and recovery times of the prepared sensors at 5 mm s-1 at different pressures under (a) 10 kPa, (b) 30 kPa, (c) 70 kPa, (d) 90 kPa.

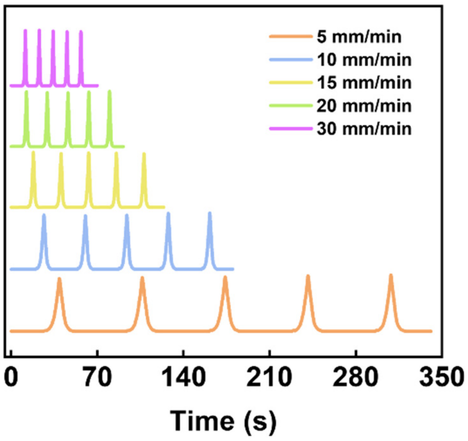

Figure S15. The I-T variation with different running speeds of the uniaxial motion controller.
